# A comparative evaluation of the effect of internet-based CME delivery format on satisfaction, knowledge and confidence

**DOI:** 10.1186/1472-6920-10-10

**Published:** 2010-01-29

**Authors:** Vernon R Curran, Lisa J Fleet, Fran Kirby

**Affiliations:** 1 Professional Development & Conferencing Services, Faculty of Medicine, Memorial University, St John's, Canada

## Abstract

**Background:**

Internet-based instruction in continuing medical education (CME) has been associated with favorable outcomes. However, more direct comparative studies of different Internet-based interventions, instructional methods, presentation formats, and approaches to implementation are needed. The purpose of this study was to conduct a comparative evaluation of two Internet-based CME delivery formats and the effect on satisfaction, knowledge and confidence outcomes.

**Methods:**

Evaluative outcomes of two differing formats of an Internet-based CME course with identical subject matter were compared. A Scheduled Group Learning format involved case-based asynchronous discussions with peers and a facilitator over a scheduled 3-week delivery period. An eCME On Demand format did not include facilitated discussion and was not based on a schedule; participants could start and finish at any time. A retrospective, pre-post evaluation study design comparing identical satisfaction, knowledge and confidence outcome measures was conducted.

**Results:**

Participants in the Scheduled Group Learning format reported significantly higher mean satisfaction ratings in some areas, performed significantly higher on a post-knowledge assessment and reported significantly higher post-confidence scores than participants in the eCME On Demand format that was not scheduled and did not include facilitated discussion activity.

**Conclusions:**

The findings support the instructional benefits of a scheduled delivery format and facilitated asynchronous discussion in Internet-based CME.

## Background

Larger numbers of physicians are using the Internet to locate and seek medical information and it has been suggested that one of its greatest benefits is as a tool for professional development [1-3]. Internet-based learning has become an increasingly popular approach to medical education [4-6] and Internet-based continuing medical education (CME) has grown steadily in the recent past [7,8]. The Internet has expanded opportunities for the provision of a flexible, convenient and interactive form of CME that has fulfilled the requirements of busy practitioners who have difficulty attending formal education sessions [9,10].

Internet-based CME has been associated with favorable outcomes across a wide variety of learners, learning contexts, clinical topics and learning outcomes [5]. According to Wearne, [8] these programs can vary in style, content, relevance, reliability, authorship and sponsorship, and hence educational quality. A variety of Internet technologies, instructional methods and presentation formats are being used to provide both asynchronous and synchronous forms of Internet-based CME [2]. Internet-based CME is commonly offered, although not exclusively, through the use of learning management systems (LMS) and web conferencing systems. A learning management system is software for delivering, tracking and managing Internet-based education and often includes features for learning assessment and online collaboration (e.g. chat, discussion board and e-mail). Web conferencing systems can facilitate synchronous presentations via the Internet. Participants are connected with other participants through their computer and can view real-time presentations while interacting with a presenter over a standard telephone line or Voice over Internet Protocol (VoIP) audio technology. Some systems also include whiteboards, chat and polling features. In a systematic review of Internet-based CME literature, Cook et al.[5] found that Internet-based instruction addresses a wide range of topics with most interventions involving tutorials for self-study or virtual patients, and over a quarter requiring online discussion with peers, instructors or both.

The main benefits of Internet-based CME include: improved access, convenience and flexibility; reduced travel expenses and time; adaptability to learning styles; just-in-time learning; and an interactive multimedia format [5,6,11,12]. Curran and Fleet's [2] review of Internet-based CME evaluation literature found that physicians are generally satisfied with it and in some instances more satisfied than with traditional CME formats. Wutoh et al. [11] also reviewed the evaluation literature and concluded that Internet-based CME is as effective in imparting knowledge as traditional formats of CME. Cook et al.'s [5] systematic review found that Internet-based learning is educationally beneficial and can achieve results similar to those of traditional instructional methods. This review also suggested that effective learning outcomes appeared to be associated with cognitive interactivity, peer discussion, on-going access to instructional materials and practice exercises [5].

It has been suggested that further research comparing Internet-based interventions against no-intervention comparison groups is of little value [5]. Further research in the field should investigate elements of Internet-based CME that could make it more effective and efficient, such as specific instructional methods, presentation formats, and approaches to implementation [5]. According to Cook et al. [5] examining how to effectively implement Internet-based instruction must involve research directly comparing different Internet-based interventions. Curran and Fleet [2] have also suggested the need to examine in greater detail the nature and characteristics of those Internet-based learning technologies, environments and systems which are most effective. There are limited comparative studies of this nature reported in the Internet-based CME literature [2,5]. In one study, Beal et al. [13] compared the effectiveness of different curriculum delivery strategies (e.g., e-mail versus web site) and duration of delivery in providing Internet-based CME. They found no significant difference in knowledge, confidence and communication by curriculum delivery strategy.

A number of other studies have examined the specific use of both asynchronous technologies (e.g., e-mail, discussion boards) and synchronous technologies (e.g., Web conferencing) for facilitating Internet-based CME and the results have been generally mixed [2]. A number of authors report findings on the effectiveness of Internet-based CME facilitated by way of electronic mail or online discussion boards, however do not compare these approaches to other Internet-based interventions [14-17]. In one study, live CME participants made very little use of either e-mail or telephone to contact faculty, however 85% of online CME participants signed on at some point in time during web conferencing sessions [6]. Guan et al. [18] examined physicians' participation in online learning discussions, perceptions of online social closeness, and barriers and motivators to participation. Lack of time and peer response were given as the main reasons for low participation in learning discussions. Weir et al. [19] also studied the effectiveness of an e-mail based discussion forum using clinical cases as stimulus material. Message postings from 27 participants were most frequent during the first of four weeks and lowest during the second. Curran et al. [20] examined the nature of the interactions and collaborative learning characteristics exhibited in Internet-based CME that included asynchronous, text-based computer discussion. The results suggested that the nature of participation consisted primarily of independent messages with a minimal amount of learner-to-learner interaction [20].

While the literature examining the use of asynchronous communications (e.g. e-mail, discussion boards) in Internet-based CME is suggestive of some limitations in its use, the principles for supporting the use of such approaches is strongly supported by adult learning theory. One theory in particular, social constructivism, views learning to be an active rather than passive endeavor. Social constructivists propose that learning is a dialogic process in which communities of practitioners engage socially in talk and activity about shared problems or tasks [21,22]. Learning occurs through engaging, incorporating and critically exploring the views of others, while new possibilities of interpretations are opened through the interaction [21]. Making meaning is the ultimate goal of constructivist learning processes [23,24], and to make meaning, constructivists believe that learners must be encouraged to articulate and reflect on what they know. Asynchronous communications are a critical component in the design of Internet-based constructivist learning environments (CLEs) as such technologies, if used effectively, can foster interaction, collaboration, and knowledge building. The communicative learning approaches which can be facilitated enable adult learners to participate in a collaborative process of building and reshaping understanding with and among their peers [25,26].

The purpose of the study described in this paper was to conduct a comparative evaluation of two differing Internet-based CME delivery strategies and the effect of a scheduled delivery format and facilitator-led asynchronous discussion instructional strategy on satisfaction, knowledge and confidence outcomes.

## Methods

Two formats of an Internet-based CME course entitled *Emergency Medicine (EM) (Trauma Cases) *have been offered via the MDcme.ca web portal (Table 1). Both formats were developed using a proprietary Internet-based learning management system. The instructional design of the Scheduled Group Learning (SGL) format was based on participation in case-based asynchronous discussions with peers and a facilitator over a scheduled delivery period, and review of online learning tutorials and resources. The SGL format was offered over a three week period and participants were required to log-in and access the course at least twice over the scheduled duration and review discussion postings. The eCME On Demand instructional format was based mainly on principles of self-directed learning. This course format was not scheduled so participants could start and finish at any time. An asynchronous discussion board was available, however the discussion was not facilitated. Participants in the On Demand format were required to complete post-assessments to receive CME credit, however there was no requirement to post messages. Both formats were designed around case-based learning principles and learning objectives and subject matter was identical. Both formats were also offered free of charge and courses were promoted through the MDcme.ca web portal, web sites of other MDcme.ca consortium partners, the MDcme.ca newsletter distributed by e-mail messaging, and by promotion at professional conferences and meeting. The SGL format was offered 9 times between February 2004 and October 2006, and On Demand was made available between June 2006 and September 2008.

**Table 1:** Internet-based CME Formats

**Format I: Scheduled Group Learning (SGL)**

case-based, asynchronous discussions with peers and a facilitator (expert)
online learning tutorials and resources
offered over a scheduled delivery period

**Format II: eCME On Demand**

asynchronous discussion board available, however discussion was not facilitated
online learning tutorials and resources
self-directed learning design
not scheduled, participants could start at any time

Pre-to-post evaluation designs were applied to both course formats. Participants were self-selecting and restricted to licensed physicians (e.g., family medicine or specialists) and postgraduate residents. Participants in both formats were asked to complete an identical participant satisfaction survey, and pre-post knowledge and pre-post confidence assessment instruments. The satisfaction survey was comprised of 10 evaluative statements rated along a 5-point Likert scale (1 = Strongly Disagree to 5 = Strongly Agree). The survey was designed to evaluate several areas, including: content; design (navigability/process); and satisfaction with online discussions and interaction. Participants were also asked to complete pre and post-tests immediately prior to and after completion of a course, respectively. The pre and post-knowledge assessment was comprised of 5 identical one-best answer (A-type) MCQ items (1 key and 3 distractors), for a maximum score of 5. The knowledge assessment was intended to measure participants' general knowledge of the subject matter covered in the courses. The pre and post-confidence assessment was comprised of 5 identical self-efficacy statements rated along a 5-point Likert scale (1 = Strongly Disagree to 5 = Strongly Agree), for a maximum score of 25. The confidence assessment was intended to measure participants' self-reported confidence in the clinical management of emergency trauma cases (e.g., *I am confident in my ability to recognize the importance of complications in elderly patients with rib fractures*; *I am confident in my ability to discuss the approach to management of a dorsal dislocation of the PIP joint*). The pre and post-assessment instruments were available on-line using the quiz tool function of the learning management system.

Ethics approval was received from the Human Investigations Committee, Memorial University of Newfoundland.

## Results

Table 2 summarizes the participant characteristics for both the SGL (N = 29) and the On Demand (N = 124) Internet-based CME formats. Groups were comparable across the majority of characteristics. The majority of participants in both formats were male (69.0% SGL vs 54.0% On Demand), family physicians (60.7% SGL vs 66.1% On Demand) and reported experience of 10 years or less (51.7% SGL vs 55.3% On Demand). The majority of participants in both formats also reported practicing in communities with a population greater than 10,000 (75.0% SGL vs 53.5% On Demand) and indicated computer skills to be of an intermediate level (69.0% SGL vs 64.2% On Demand). Pearson Chi Square analysis indicated that the On Demand group had a significantly higher proportion of participants reporting previous online CME experience (p = .012).

**Table 2:** Participant Characteristics

		SGL		On Demand		Pearson Chi Square	
		
		N	%	N	%	df	**Sig**.
Gender	Male	20	69.0%	67	54.0%		
	Female	9	31.0%	57	46.0%	1	.144
							
Physician Type	Family Physician	17	60.7%	82	66.1%		
	Other Specialist	7	25.0%	22	17.1%	3	.823
	Resident	2	7.1%	8	6.5%		
	Other	2	7.1%	12	9.7%		
							
Years of Experience	0-5 years	8	27.6%	37	30.1%		
	6-10 years	7	24.1%	31	25.2%		
	11-15 years	8	27.6%	20	16.3%		
	16-20 years	1	3.4%	10	8.1%	6	.558
	21-25 years	2	6.9%	18	14.6%		
	26-30 years	2	6.9%	6	4.9%		
	> 30 years	1	3.4%	1	0.8%		
							
Size of Population	< 5,000	4	14.3%	33	27.0%		
That Depends on	5,000-9,999	3	10.7%	24	19.7%	2	.111
Participant for Primary Care	> 10,000	21	75.0%	65	53.5%		

Computer Skills	Beginner	3	10.3%	11	8.9%		
	Intermediate	20	69.0%	79	64.2%	2	.788
	Expert	6	20.7%	33	26.8%		
							
Previous Experience with Online CME	Yes	16	55.2%	96	78.0%	1	.012
	No	13	44.8%	27	22.0%		

Table 3 summarizes satisfaction ratings across the two formats. A total of 28 respondents from the SGL format completed the satisfaction survey while 124 from the eCME On Demand format responded. The ratings suggest respondents were very satisfied with the Internet-based instruction, regardless of format. Participants in both formats reported very positive overall mean scores for *"The content was applicable to my practice" *(*M *= 4.57 SGL vs *M *= 4.06 On Demand) and *"I would participate in another CME course offering of this type" *(*M *= 4.57 SGL vs *M *= 4.34 On Demand). An independent t-test analysis indicated that participants in the SGL format reported significantly higher mean ratings for items related to learning needs (p = .038) and clarity of content (p = .028) at the p < .05 probability level.

**Table 3:** Satisfaction Ratings by Format*

Survey Questions	Format	N	Mean Response	SD	**Sig**.
The content addressed my learning needs.	SGL On Demand	28 122	4.46 3.94	.576 1.180	.038
The content was applicable to my practice.	SGL On Demand	28 122	4.57 4.06	.504 1.166	.065
The content was clear and easy to understand.	SGL On Demand	28 121	4.61 3.98	.685 1.235	.028
This online course was easy to use.	SGL On Demand	28 121	4.14 3.81	1.044 1.142	.550
The pages were clearly laid out.	SGL On Demand	28 119	4.43 4.10	.690 1.020	.220
I received adequate help with technical problems.	SGL On Demand	14 83	3.29 3.67	.914 1.083	.209
Participating in the discussions enhanced my understanding of the content.	SGL On Demand	27 107	4.07 3.89	1.035 .955	.158
Being able to communicate with colleagues was helpful.	SGL On Demand	25 91	3.88 3.56	.927 1.056	.197
I would participate in another CME course offering of this type.	SGL On Demand	28 117	4.57 4.34	.790 1.101	.342
I would recommend this course to others.	SGL On Demand	28 123	4.57 4.17	.690 1.092	.063

* Satisfaction survey was comprised of 10 evaluative statements rated along a 5-point Likert scale (1 = Strongly Disagree to 5 = Strongly Agree).

Table 4 summarizes the overall mean pre and post-knowledge assessment scores. A total of 13 participants in the SGL format completed both pre and post-knowledge assessments while 74 participants from the On Demand format completed both assessments. Participants in the SGL format reported an overall mean pre-knowledge score of 2.38 and a post-knowledge score of 4.08. Participants in the On Demand format reported an overall mean pre-knowledge score of 1.72 and a post-knowledge score of 3.08. A paired samples t-test analyses indicated a significant pre to post-knowledge increase (p = .000) for both course formats at the p < .05 probability level.

**Table 4:** Overall Pre to Post-Knowledge Change

Format		N	Mean Score (out of 5)	SD	**Sig**.
SGL	Pre-CME	13	2.38	.768	.000
	Post-CME	13	4.08	1.115	
On Demand	Pre-CME	74	1.72	.958	.000
	Post-CME	74	3.08	.962	

Table 5 summarizes the results of an independent samples t-test comparing the pre and post-knowledge assessment results between formats. Participants in the SGL format performed significantly higher at the p < .05 probability level on both the pre (p = .012) and post-knowledge assessment (p = .008) than participants in the On Demand format.

**Table 5:** Pre to Post-Knowledge Change Between Formats

Knowledge Assessment		N	Mean Score (out of 5)	SD	**Sig**.
Pre-CME	SGL	13	2.38	.768	.012
	On Demand	74	1.72	.958	
Post-CME	SGL	13	4.08	1.115	.008
	On Demand	74	3.08	.962	

Table 6 summarizes the overall mean pre and post-confidence assessment scores. A total of 13 participants completed both the pre and post-confidence assessments in the SGL format while 73 participants completed both assessments in the On Demand format. Participants in the SGL format reported an overall mean pre-confidence score of 17.23 and a post-confidence score of 21.62. Participants in the On Demand format reported an overall mean pre-confidence score of 18.51 and a post-confidence score of 17.81. A paired samples t-test analysis indicated that only participants in the SGL format reported a significant increase (p = .005) in pre to post-confidence scores at the p < .05 probability level.

**Table 6:** Overall Pre to Post-Confidence Change

Format		N	Mean Score (out of 25)	SD	**Sig**.
SGL	Pre-CME	13	17.23	2.127	.005
	Post-CME	13	21.62	5.378	
On Demand	Pre-CME	73	18.51	4.634	.505
	Post-CME	73	17.81	7.501	

Table 7 summarizes the results of an independent samples t-test comparing the pre and post-confidence assessment results between formats. Participants in the SGL format reported significantly higher post-confidence scores (p = .039) than participants in the On Demand format at the p < .05 probability level.

**Table 7:** Pre to Post-Confidence Change Between Formats

Confidence Assessment		N	Mean Score (out of 25)	SD	**Sig**.
Pre-CME	SGL	13	17.23	2.127	.120
	On Demand	73	18.51	4.634	
Post-CME	SGL	13	21.62	5.378	.039
	On Demand	73	17.81	7.501	

## Discussion

The findings indicate that an Internet-based CME format involving facilitated asynchronous discussion activity and a delivery schedule over a three week time period resulted in more positive learning outcomes in comparison to an Internet-based CME format which was not based on a learning schedule and did not involve facilitated discussion activity. Participants in the SGL format reported significantly higher mean satisfaction ratings for items related to Internet-based CME *"meeting learning needs" *and *"content being clear and easy to understand"*. Participants in this format also performed significantly higher on a post-knowledge assessment and reported significantly higher post-confidence scores than participants in the On Demand format. The higher level of online CME experience reported by participants in the On Demand format did not appear to affect learning outcomes of this group.

A primary limitation of this exploratory study is related to generalization. This was a study of the use of particular Internet-based learning technologies, delivery methods and learning approaches. As well, the subject matter of the courses was related to a very specific clinical area. The results must be viewed and interpreted in this context. 	The two course formats were offered at different time periods and it is possible that historical factors may have influenced the outcome measures and/or participants' knowledge and attitudes may have changed during this time. Another limitation to the study was the difference in participant numbers between the two course formats. Registration for the SGL format was limited to 20 participants per course offering in order to foster enhanced facilitator interaction with participants. There was no limitation in registration for the On Demand course format. It is possible that the difference in the number of registrants between the course formats may have influenced the overall mean scores reported in the results. However, a comparison of participant characteristics did not indicate any significant differences between the study groups in terms of gender, physician type, years of experience, population size and computer skills. As well, a large number of participants did not complete both pre and post-knowledge and confidence assessments. The study results only represent matched scores for participants completing both pre and post assessments. Participants were not required to complete both pre and post assessment instruments to receive course credit.

Basing course completion around a schedule and supplementing instruction with a case-based asynchronous discussion board activity may have been key motivational factors in the SGL format and hence contributed to the positive learning outcomes. This finding is supported in previous research by Fordis et al. [6] who found that Internet-based CME participants often completed learning activities over several sessions. These researchers also reported that exposure to an educational activity (e.g., online discussion) combined with multi-session use of online materials may indicate an advantage of sequential reinforcement with Internet-based education [6]. It is likely that the increased interactivity and collaborative learning experiences afforded by an asynchronous discussion activity led by a facilitator, and subsequent opportunities for reflection on practice because of the scheduled nature of the learning, may have contributed to greater learning benefits.

The role of the facilitator in the SGL format was also not examined in detail and it is likely that the way in which the discussion board activities were facilitated may have influenced the learning outcomes for the participants in this format. Previous research does suggest that the level of asynchronous discussion participation by both facilitators and other participants in Internet-based CME is related to individual learner participation [27]. The findings of this study do highlight the significance of facilitated discussion and the important role of facilitation in fostering positive learning outcomes in Internet-based CME.

This study focused mainly on the effect of scheduled learning and asynchronous learning activities, however it is possible that synchronous instructional and communicative interaction facilitated through the use of real-time Web conferencing systems could also afford similar benefits. Future research should examine the comparative effectiveness and benefits of asynchronous versus synchronous interaction on similar learning outcome measures. A useful area for further research would also be to explore how "blended approaches" to Internet-based CME delivery, combining both asynchronous and synchronous formats, might be used effectively. It would also be beneficial for future studies to examine the effect of participation in differing Internet-based formats on subsequent physician practices and behavior. How can Internet-based CME and associated technologies be used effectively to not only foster meaningful learning, but also support and foster knowledge transfer and practice change?

## Conclusions

The purpose of this study was to conduct a comparative evaluation of two differing Internet-based CME delivery strategies and the effect of a scheduled delivery format and facilitator-led asynchronous discussion instructional strategy on satisfaction, knowledge and confidence outcomes. A Scheduled Group Learning (SGL) Internet-based CME format facilitated learning that incorporated participation in case-based asynchronous discussions with peers and a facilitator over a scheduled delivery period. An eCME On Demand Internet-based CME format was not scheduled so participants could start and finish at any time. The results of the study indicate that the SGL format resulted in more positive learning outcomes in comparison to the On Demand format. Participants in the SGL format reported significantly higher mean satisfaction ratings, performed significantly higher on a post-knowledge assessment and reported significantly higher post-confidence scores. The findings from this study support the instructional benefits of a scheduled delivery format and facilitated asynchronous discussion in the delivery of Internet-based CME.

## Competing interests

The authors declare that they have no competing interests.

## Authors' contributions

VC led the conceptualization of the study design, participated in data analysis and led the preparation of the manuscript. LF led data collection and analysis, and participated in drafting the manuscript. FK helped to draft the manuscript. All authors read and approved the final manuscript.

## Pre-publication history

The pre-publication history for this paper can be accessed here:

http://www.biomedcentral.com/1472-6920/10/10/prepub
